# A photodistributed rash in a patient on apixaban

**DOI:** 10.1016/j.jdcr.2024.11.005

**Published:** 2024-11-26

**Authors:** Benjamin Donald Henson, Rachel B. Lee

**Affiliations:** a Virginia Commonwealth University School of Medicine, Richmond, Virginia; b Department of Dermatology, VCU Health System, Richmond, Virginia

**Keywords:** ANA, anticoagulation, Anti-histone antibodies, apixaban, autoimmune, cutaneous lupus, direct oral anticoagulants, drug induced lupus, histology, histopathology, subacute cutaneous lupus erythematosus

## Case vignette

**Fig 1:**
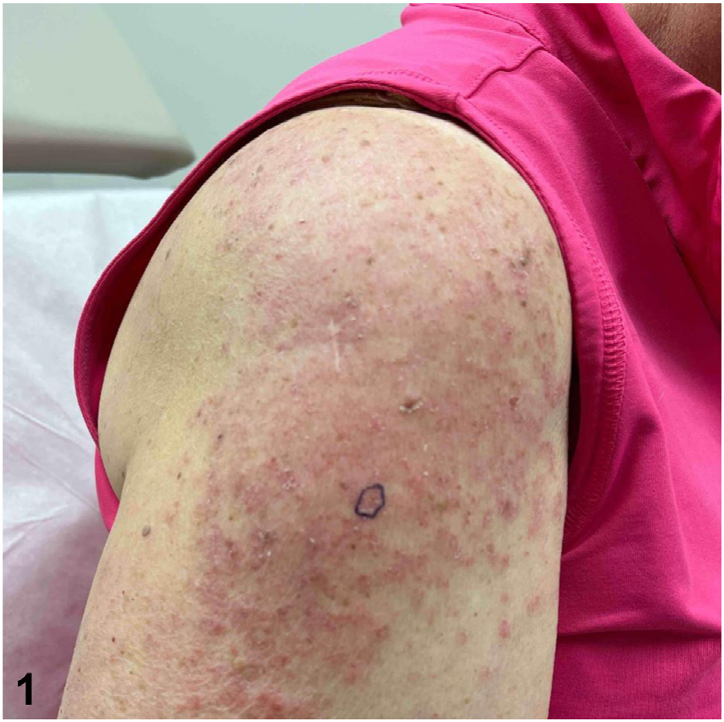

**Fig 2:**
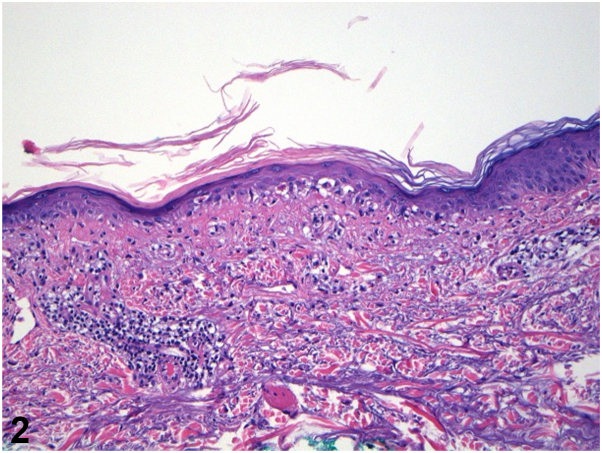

**Fig 3:**
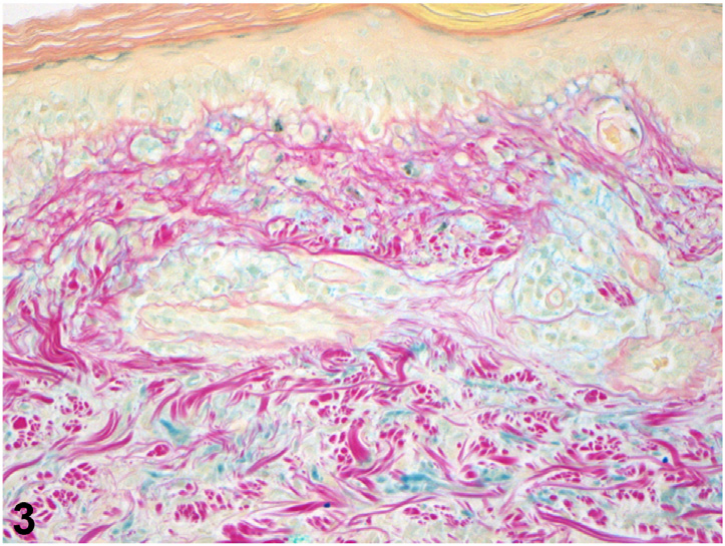

A 63-year-old female with recent diagnosis of atrial fibrillation presented with a 1 month history of pruritic rash. Apixaban was started 6 weeks prior to presentation after cardiac ablation. She was using triamcinolone ointment without improvement. Exam showed photodistributed erythematous coalescing scaly papules on the vertex scalp, shoulders, and upper arms, with sharp demarcation along shirt line (Fig 1). Shave biopsy showed interface dermatitis, dermal mucin, and superficial dermal perivascular lymphocytic infiltrate (Figs 2 and 3). Lab testing was notable for anti-histone antibody 3.7U (strong positive), elevated liver enzymes, normal complete blood count, and negative anti-nuclear antibody (ANA), anti-SSA, and SSB antibodies.

**Question 1: What is the most likely diagnosis for the patient's skin condition?**

A. Psoriasis
B. Discoid lupus erythematosus
C. Dermatomyositis
D. Jessner’s lymphocytic infiltrate of the skin
E. Subacute cutaneous lupus erythematosus (SCLE)

**Answers:**

A. Psoriasis – Incorrect. Psoriasis typically presents with well-defined erythematous plaques covered with silvery scales, commonly involving extensor surfaces like elbows and knees. It typically exhibits parakeratosis, acanthosis, and Munro microabscesses.
B. Discoid lupus erythematosus – Incorrect. Discoid lupus erythematosus presents with discoid lesions, often with adherent scales, and can cause scarring. It typically lacks the widespread photodistribution and histology findings including superficial and deep perivascular lymphocytic infiltrate.
C. Dermatomyositis – Incorrect. Dermatomyositis can have a similar photodistribution and erythematous papules, but it is often associated with proximal muscle weakness, heliotrope rash, and Gottron's papules. Histopathologic findings however can include interface dermatitis and dermal mucin deposition.
D. Jessner’s lymphocytic infiltrate of the skin – Incorrect. Jessner’s lymphocytic infiltrate of the skin can present photodistributed papular rash with histology showing dermal lymphocytic infiltrate. However, dermal mucin and interface dermatitis along with a strong anti-histone antibody positivity make another diagnosis more likely.
E. Subacute cutaneous lupus erythematosus (SCLE) – Correct. SCLE typically presents with photodistributed erythematous scaly papules and specific histopathologic features including interface dermatitis, mucin deposition, and superficial perivascular lymphocytic infiltrate.^1^

**Question 2: What is the recommended management strategy for this patient?**

A. Discontinuation of apixaban in consultation with cardiologist, topical corticosteroids, and sun protection
B. Oral hydroxychloroquine
C. Methotrexate
D. Discontinuation of apixaban and initiation of rivaroxaban
E. Systemic corticosteroids

**Answers:**

A. Discontinuation of apixaban in consultation with cardiologist, topical corticosteroids, and sun protection – Correct. Initial management of SCLE involves topical corticosteroids for localized lesions and strict sun protection measures to prevent exacerbation of the photosensitive rash. Adjusting medications known to induce SCLE, if possible, is also recommended.
B. Oral hydroxychloroquine – Incorrect. While hydroxychloroquine is a standard treatment for SCLE, it is often used in combination with topical corticosteroids initially, especially for mild cases. However, it is not typically the first-line therapy without adjunctive topical treatment.
C. Methotrexate – Incorrect. Methotrexate can be considered in more severe or refractory cases of SCLE but is not typically first-line therapy. It may be used as a steroid-sparing agent or in combination with other treatments.
D. Discontinuation of apixaban and initiation of rivaroxaban – Incorrect. Both apixaban and rivaroxaban are factor Xa inhibitors, or direct oral anticoagulants. This class of medication has been rarely reported as culprits of drug induced lupus and there is insufficient data to suggest switching to a medication within the same class is safe or effective.
E. Systemic corticosteroids – Incorrect. Systemic corticosteroids are reserved for more severe cases or when topical treatments are inadequate. This patient was transitioned to ultra-potent topical steroids

**Question 3: Which of the following has *not* been associated with increased risk of progression from cutaneous lupus erythematosus (CLE) to systemic lupus erythematosus (SLE)?**

A. Presence of interface dermatitis on histology
B. Positive ANA
C. Younger age of onset
D. Positive anti-SSA antibody
E. Hematologic abnormalities

**Answers:**

A. Presence of interface dermatitis on histology – Correct. While interface dermatitis is a common feature in CLE, histologic findings have *not* been linked to an increased risk of progression to SLE.
B. Positive ANA – Incorrect. Positive serology and high titers of ANAs have been well documented to increase risk of progression from CLE to SLE.^2^
C. Younger age of onset – Incorrect. A younger age of onset of CLE has been shown to increase risk of progression to SLE. Conversely an older age of onset has shown to decrease risk.^2^
D. Positive anti-SSA antibody – Incorrect. Positive anti-SSA antibodies have been associated with an increased risk of progression.^3^
E. Hematologic abnormalities – Incorrect. Hematologic abnormalities such as leukopenia and anemia have been shown to increase risk of progression.^4^

## Conflicts of interest

None disclosed.
